# Evaluation of thermo-chemical conversion temperatures of cannabinoid acids in hemp (*Cannabis sativa* L.) biomass by pressurized liquid extraction

**DOI:** 10.1186/s42238-021-00098-6

**Published:** 2021-08-31

**Authors:** Kenneth J. Olejar, Chad A. Kinney

**Affiliations:** 1 Chemistry Department, Colorado State University — Pueblo, 2200 Bonforte Blvd, Pueblo, CO 81001 USA; 2 Institute for Cannabis Research, Colorado State University — Pueblo, 2200 Bonforte Blvd, Pueblo, CO 81001 USA

**Keywords:** Cannabinoid acids decarboxylation

## Abstract

**Background:**

Cannabinoids are increasingly becoming compounds of medical interest. However, cannabis plants only produce carboxylated cannabinoids. In order to access the purported medical benefits of these compounds, the carboxylic acid moiety must be removed. This process is typically performed by heating the plant material or extract; however, cannabinoids being thermolabile can readily degrade, evaporate, or convert to undesired metabolites. Pressurized liquid extraction (PLE) operates using a pseudo-closed system under pressure and temperature. While pressure is maintained at 11 MPa, temperature can be varied from ambient to 200 °C.

**Methods:**

Temperatures were evaluated (80 to 160 °C) using PLE for the thermo-chemical conversion of cannabinoid acids utilizing water as the solvent in the first step of extraction with subsequent extraction with ethanol. Optimum temperatures were established for the conversion of 6 cannabinoid acids to their neutral cannabinoid forms. Cannabinoid acid conversion was monitored by HPLC.

**Results:**

The use of PLE for thermo-chemical decarboxylation has resulted in a rapid decarboxylation process taking merely 6 min. The temperatures established here demonstrate statistically significant maxima and minima of cannabinoids and their parent cannabinoid acids. One-way ANOVA analysis shows where individual cannabinoids are statistically different, but the combination of the maxima and minima provides temperatures for optimum thermo-chemical conversion. CBC, CBD, CBDV, and CBG have an optimum temperature of conversion of 140 °C, while THC was 120 °C for 6 min.

**Discussion:**

Decarboxylation of cannabinoid acids is necessary for conversion to the bioactive neutral form. The pseudo-closed chamber of the PLE makes this an ideal system to rapidly decarboxylate the cannabinoid acids due to pressure and temperature, while minimizing loss typically associated with conventional thermal-decarboxylation. This study established the optimum temperatures for thermo-chemical conversion of the cannabinoid acids in water and provides the groundwork for further development of the technology for industrial scale application.

## Background

With the passage of the 2018 Farm Bill, hemp cultivation has become more prevalent with an increase from 37,122 acres in 2017 to 310,721 acres in 2019 (Sterns 2019). While some of the crop is used for fibers and seed oil, a large amount is used for cannabinoid isolation. While the extraction process is well established, the extracted cannabinoids are in their acidic forms unless decarboxylation in an oven is done on the biomass prior to extraction or decarboxylation of the oleoresin is done to obtain the neutral cannabinoids for the majority of the purported health benefits, with the prior being more prevalent (Russo 2018).

The decarboxylation process is known to result in evaporation of cannabinoids and due to the thermolability can result in cannabinoid degradation and unwanted metabolites (Moreno et al. 2020). Therefore, having optimum temperatures for the conversion is essential to maximizing conversion and minimizing loss. However, there is no one temperature that fits all cannabinoids, so a single or group of cannabinoids with similar decarboxylation temperatures must be targeted.

While decarboxylation of cannabinoids is typically done in an oven, there are reports of other compounds being decarboxylated using a thermo-chemical approach (Mundle and Kluger 2009; Mundle et al. 2010; Vandersteen et al. 2012). These processes occur in a solvent capable of donating a hydrogen atom to the carboxylic acid moiety, which aids in its cleavage from the cannabinoid. One such solvent is water and its use in decarboxylation has been extensively studied (Mundle and Kluger 2009; Mundle et al. 2010; Vandersteen et al. 2012; Hossain et al. 2017; Glein et al. 2020).

Conventional extraction processes are common in the cannabinoid industry (WHO 2018). The techniques are simple and do not require large upfront costs to get started. The extractions can also be combined with various pretreatments, such as microwave or ultrasound, or it can be done in soxhlet (Fiorini et al. 2020; Kenari and Dehghan 2020). A modernized alternative for soxhlet is pressurized liquid extraction (PLE), where solvent extraction occurs at increased pressure and temperature to facilitate a rapid extraction. However, this process still has the limitation of requiring hemp biomass to be decarboxylated prior to extraction or the oleoresin must undergo decarboxylation to obtain neutral cannabinoids.

To combat the need for decarboxylation to occur during a separate step, it was hypothesized that thermo-chemical conversion of acidic cannabinoids to their neutral counterparts could be done during the extraction process using PLE. Furthermore, it was speculated that this process could be accomplished using water as the solvent, thereby minimizing the cannabinoid loss as a result of their low to negligible solubility in water (Pertwee 2009). Consequently, the temperatures necessary to decarboxylate the common cannabinoids in hemp biomass using the PLE system were examined.

## Methods

### Materials

Reagents used in the study were HPLC grade. Methanol, ethanol, and Ottawa sand (20–30 mesh) were obtained from Thermo Fisher Scientific (Waltham, MA, USA). Ottawa sand was ashed at 400 °C for 4 h prior to use. Formic acid was obtained from Fluka (St. Louis, Mo, USA). Ultra-pure water (18MΩ-cm) was obtained from a Barnstead NanoPure Infinity Ultrapure Water System (Thermo Fisher Scientific). Ultra-high purity nitrogen was obtained from Airgas (Pueblo, CO, USA).

Thirteen cannabinoid standard solutions, cannabinchromene (CBC), cannabichromenic acid (CBCA) cannabidiol (CBD), cannabidiolic acid (CBDA), cannabigerol (CBG), cannabigerolic acid (CBGA) cannabinol (CBN), cannabidivarin (CBDV), cannabidivarinic acid (CBDVA), Δ^9^-tetrahydrocannabinol (THC), Δ^9^-tetrahydrocannabinolic acid (THCA), Δ^9^-tetrahydrocannabivarin (THCV), and Δ^9^-tetrahydrocannabivarinic acid (THCVA), at a concentration of 1.0 mg mL^−1^ in methanol or acetonitrile were purchased from Cerilliant (San Antonio, TX, USA). A stock solution of the eleven cannabinoids (CBC, CBD, CBDA, CBDV, CBG, CBGA, CBN, THC, Δ^8^-THC, THCA, THC) at 100 mg L^−1^ was prepared in HPLC grade methanol from a commercial 1.0 mg mL^−1^ solution (Cayman Chemical Company, Ann Arbor, MI, USA).

### Industrial hemp biomass

Industrial hemp biomass consisting of leaf and small inflorescences was supplied by Sacred Giftz (Longmont, CO, USA). The biomass was comprised of the cannabis hybrid Boax, which is 50% sativa and 50% indica being derived from *Cannabis sativa ssp. sativa* Hindu Kush and *Cannabis sativa ssp. indica* Otto II strains resulting in the Boax species producing inflorescences containing 18 to 20% CBDA and 0 to 1% THC. The plants were grown outdoors in Colorado planted in a south to north configuration with plants 1 m on center and rows 1.2 m apart. The fields were planted in 11 June 2019 with harvest occurring 25 September to 5 October. The biomass received was passed through screens to remove seeds and stalk. The remaining material was then ground to pass through a 1.18 mm screen and stored at 4 °C in sealed bags until use.

### Thermo-chemical conversion

Pressurized liquid extraction (PLE) operates at a pressure of 11.0 MPa. Temperature and time are variables that can be utilized to maximize the processes. Temperatures were examined from 80 to 160 °C and utilizing a static cycle time of 3 min. A 10-mL stainless steel cell containing a glass fiber filter (Thermo Fisher Scientific), 1.5 g of Ottawa sand, 1.0 g of hemp biomass, and remaining cell space filled with Ottawa sand. The experiments were carried out on a Dionex ASE-350 operated by Chromeleon software, version 7.2 SR5 (Thermo Fisher Scientific).

### HPLC

Cannabinoid quantification was made by liquid chromatography on a Thermo Scientific Dionex UltiMate 3000 HPLC system (Thermo Fisher Scientific) equipped with a temperature-controlled autosampler (WPS 3000TSL Analytical) and a diode array detector with multiple wavelength detection (DAD 3000 and MWD 3000). The system was controlled using Chromeleon 7.2 software, version 7.2 SR5 (Thermo Fisher Scientific).

Chromatographic separation of cannabinoids was accomplished using an Accucore aQ C18 Polar Endcapped column, I.D. 100 mm × 2.1 mm, particle size 2.6 μm (Thermo Fisher Scientific) maintained at 50.0 °C. To achieve separation a gradient was employed consisting of mobile phase A, 0.1% formic acid in water, and mobile phase B, 0.1% formic acid in methanol. The gradient started at 62% B, increasing to 66% B at 13.75 min, followed by an increase to 80% B at 20 min. This was maintained for 4 min before returning to 62% B and equilibrating for 3 min. The total runtime was 24 min. The pump maintained a flow rate of 0.45 ml/min and a 2.0 μL injection was used. The wavelengths of 210 and 220 nm were monitored for analyte presence.

Integration occurred using the Chromeleon software. Cannabinoids in extracts were verified by comparison to the retention time and the UV spectra of the pure cannabinoid standards. A four-point standard curve (5, 10, 50, 100 mg L^−1^) was used to quantify the cannabinoids (Table 1).

**Table 1:** Cannabinoid analysis by HPLC: calibration curves, limits of detection, and limits of quantification

Cannabinoid	Calibration range (mg/L)	***R***^**2**^	LOD	LOQ
CBC	5.0–100	0.9999	0.436	2.51
CBCA	5.0–100	0.9982	0.418	2.40
CBD	5.0–100	0.9994	0.481	2.78
CBDA	5.0–100	0.9925	0.556	3.24
CBDV	5.0–100	0.9997	0.474	2.74
CBDVA	5.0–100	0.9997	0.560	3.26
CBG	5.0–100	0.9999	0.401	2.29
CBGA	5.0–100	0.9996	0.482	2.78
CBN	5.0–100	0.9942	0.434	2.49
THC	5.0–100	0.9963	0.654	3.83
THCA	5.0–100	0.9997	0.547	3.18
THCV	5.0–100	0.9996	0.505	2.92
THCVA	5.0–100	0.9999	0.624	3.65

*LOD* limit of detection, *LOQ* limit of quantification, *n* = 10

### Data analysis

Statistical analysis of individual cannabinoids at each temperature was performed with JMP, version 13.2.0, (SAS Institute, Cary, NC, USA) using one-way analysis of variance (ANOVA) with Tukey-honestly significant difference (HSD) post hoc testing.

## Results

**Fig. 1:**
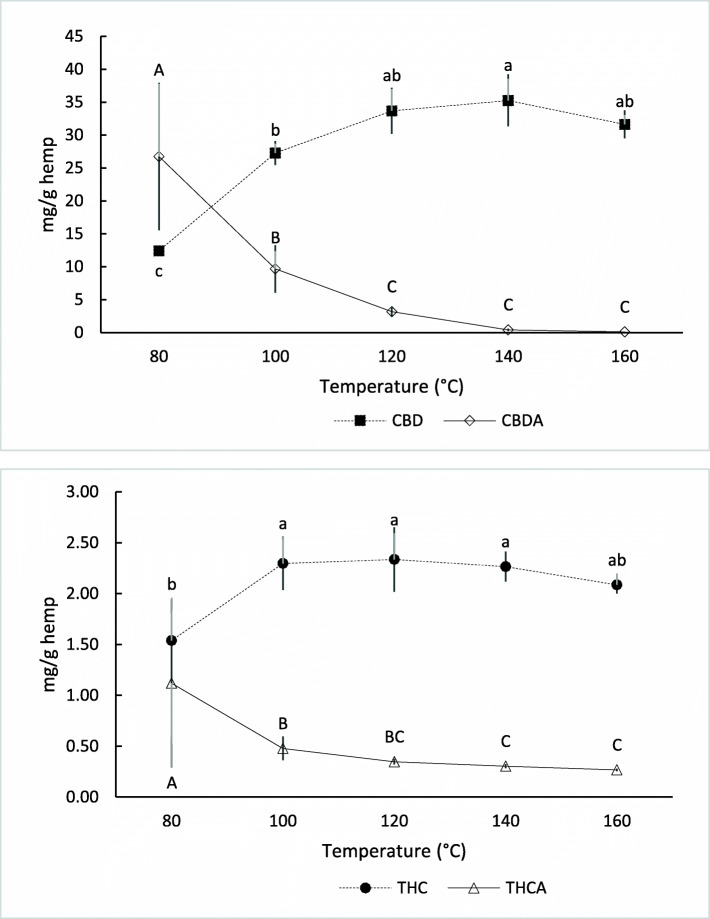
Comparative concentrations of two common cannabinoids undergoing thermo-chemical conversion. **A** CBDA to CBD and **B** THCA to THC

Table 2 highlights the various thermo-chemical conversion temperatures required to achieve maximal content of neutral cannabinoids and minimal content of acidic cannabinoids, with Fig. 1 highlighting the conversion of CBDA and THCA to CBD and TH, respectively. Statistically significant differences between the temperatures are observed for each cannabinoid with the maxima and minima occurring at different temperatures for each corresponding cannabinoid. At 100 °C, CBC, CBG, and THC had statistically the highest concentrations; however, the corresponding statistical minima occurred at 120 °C for CBCA and CBGA, and 140 °C for THCA, respectively. At 120 °C CBDV had its maxima and the corresponding CBDVA minima was at 140 °C. Cannabidiol (CBD) was greatest at 140 °C and CBDA was least at 120 °C.

**Table 2:** Thermo-chemical conversion temperature’s impact on cannabinoid extraction content expressed as mg/g hemp

	80 °C	100 °C	120 °C	140 °C	160 °C
**CBC**	1.94 ± 0.33 b	3.82 ± 0.50 a	4.41 ± 0.31 a	4.33 ± 0.14 a	4.10 ± 0.47 a
**CBCA**	2.92 ± 0.49 a	1.08 ± 0.10 b	0.426 ± 0.026 c	0.160 ± 0.035 c	0.174 ± 0.114 c
**CBD**	12.4 ± 1.8 c	27.3 ± 3.5 b	33.7 ± 3.9 ab	35.3 ± 2.1 a	31.6 ± 2.1 ab
**CBDA**	26.7 ± 3.6 a	9.68 ± 0.63 b	3.18 ± 0.16 c	0.425 ± 0.037 c	0.098 ± 0.015 c
**CBDV**	0.132 ± 0.020 c	0.201 ± 0.027 b	0.262 ± 0.016 a	0.292 ± 0.003 a	0.263 ± 0.028 a
**CBDVA**	0.321 ± 0.028 a	0.140 ± 0.009 b	0.059 ± 0.004 c	0.014 ± 0.001 d	0.004 ± 0.000 d
**CBG**	0.758 ± 0.146 b	1.58 ± 0.19 a	1.85 ± 0.14 a	1.90 ± 0.05 a	1.83 ± 0.24 a
**CBGA**	1.33 ± 0.21 a	0.574 ± 0.046 b	0.158 ± 0.015 c	0.101 ± 0.115 c	0.041 ± 0.005 c
**CBN**	0.032 ± 0.013 b	0.073 ± 0.023 a	0.080 ± 0.008 a	0.090 ± 0.002 a	0.084 ± 0.011 a
**THC**	1.54 ± 0.26 b	2.30 ± 0.32 a	2.33 ± 0.15 a	2.27 ± 0.08 a	2.09 ± 0.29 ab
**THCA**	1.12 ± 0.12 a	0.478 ± 0.028 b	0.348 ± 0.019 bc	0.303 ± 0.012 c	0.267 ± 0.036 c

Significance determined by one-way ANOVA with Tukey-HSD *post hoc* testing (*P* < 0.05) and is designated by different letter in each row

CBD and THC begin to show degradation effects with decreased quantities at 160 °C. Other cannabinoids, CBD and CBDV, begin to show the trend of degradation at this same temperature. CBN is the primary degradation product of THC shows a maxima at 100 °C. While the temperature of 160 °C demonstrates the degradation impacts of temperature on individual cannabinoid quantities, the monitoring of CBN shows that these processes occur much sooner and therefore 100% efficient conversion is not possible.

## Discussion

While statistical analysis of each cannabinoid provides insights into statistical differences a combined approach of maxima and minima must be examined for the determination of the optimum temperature. Even this must be carefully examined due to the thermolability of the compounds. As such, decreases in the acidic form amounts should be examined for corresponding increases in the neutral form concentrations. By utilizing these two methods a clearer picture of the optimum temperature can be established.

**Scheme 1:**
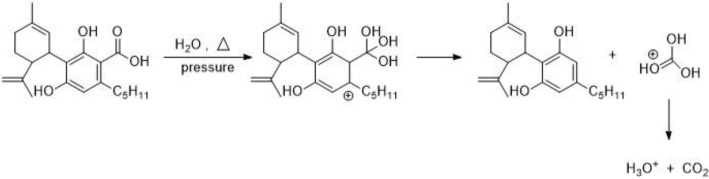
Proposed thermo-chemical conversion of representative cannabinoid, cannabidiolic acid to cannabidiol

The thermo-chemical conversion of the cannabinoid acids is proposed to proceed by Scheme 1. In Scheme 1, the proposed mechanism involves the formation of carbon dioxide with water transporting a proton the cannabinoid. This scheme is supported by Mundle and Kluger (2009), Mundle et al. (2010) were they examined the hydrolytic decarboxylation of carboxylic acids resulting in the formation of protonated carbonic acid, which decomposed to carbon dioxide and water. This mechanism was further supported by Vandersteen et al. (2012) with indolecarboxylic acid.

Thermal-decarboxylation occurs via the release of carbon dioxide. Unfortunately, the application of heat results in the degradation of thermolabile cannabinoids and evaporation (Moreno et al. 2020). As such, the combination of heat and pressure in combination with water in the PLE system facilitates the rapid decarboxylation of the cannabinoid acids. The thermo-chemical conversion of the cannabinoids results in decreased degradation of the cannabinoids as evidenced by the minor production of CBN.

Thermal-decarboxylation is reported to have losses of 20% or more through evaporation of the cannabinoid and degradation (Moreno et al. 2020; Wang et al. 2016); however, thermo-chemical conversion has losses of 5% or less. This represents an increase of 15% in cannabinoids by using thermo-chemical conversion. Additionally, thermal-decarboxylation can take up to 2 h (Moreno et al. 2020; Citti et al. 2018; Grijó et al. 2018; Veress et al. 1990; Wang et al. 2016). This is significantly greater than the 6 min required for thermo-chemical conversion.

## Conclusions

These experiments have resulted in the optimum temperatures for PLE thermo-chemical decarboxylation. When combined with solvent extraction, it provides a quick and effective means to obtain neutral cannabinoids in a single cell process. Utilization of PLE for decarboxylation and extraction can reduce energy costs, time, and the quantities of released greenhouse gasses like carbon dioxide.

## Data Availability

The datasets used and/or analyzed during the current study are available from the corresponding author on reasonable request.

## Abbreviations

PLE: Pressurized liquid extraction
CBC: Cannabinchromene
CBCA: Cannabichromenic acid
CBD: Cannabidiol
CBDA: Cannabidiolic acid
CBG: Cannabigerol
CBGA: Cannabigerolic acid
CBN: Cannabinol
CBDV: Cannabidivarin
CBDVA: Cannabidivarinic acid
Δ^8^-THC: Δ^8^-Tetrahydrocannabinol
THCA: Δ^9^-Tetrahydrocannabinolic acid
THC: Δ^9^-Tetrahydrocannabinol
